# Pain, sleep, fatigue, and health-related quality of life in pediatric sickle cell disease: a serial multiple mediator analysis

**DOI:** 10.3389/fpain.2025.1725113

**Published:** 2026-01-09

**Authors:** James W. Varni

**Affiliations:** 1 Department of Pediatrics, College of Medicine, Texas A&M University, College Station, TX, United States; 2 Department of Landscape Architecture and Urban Planning, College of Architecture, Texas A&M University, College Station, TX, United States

**Keywords:** sickle cell disease, pain, sleep, fatigue, health-related quality of life, PedsQL

## Abstract

**Background:**

Pediatric patients with sickle cell disease (SCD) experience a high level of pain, which is often chronic and recurrent and results in impaired health-related quality of life (HRQOL). Patients with SCD also report a significant degree of sleep disturbance and fatigue associated with their chronic disease. The objective of the present study is to investigate the effects of pain, sleep disturbance, and general fatigue in a serial (sequential) multiple mediator model analysis predicting overall generic HRQOL in pediatric patients with SCD from their perspective.

**Methods:**

The Pain Scale from the PedsQL Sickle Cell Disease Module, General Fatigue Scale and Sleep Disturbance Item from the PedsQL Multidimensional Fatigue Scale, and the PedsQL 4.0 Generic Core Scales were completed in a multisite national study by 227 pediatric patients with SCD aged 5–18 years. Hierarchical multiple regression and serial multiple mediator model analyses were conducted to test the percent variability accounted for and the mediating effects of sleep disturbance and general fatigue in the association between SCD pain and generic HRQOL.

**Results:**

Pain predictive effects on generic HRQOL were serially mediated by sleep disturbance and general fatigue. In a hierarchical multiple regression analysis controlling for age and sex, pain, sleep disturbance, and general fatigue accounted for 63% of the variance in pediatric patient–reported generic HRQOL (*P* < 0.001), demonstrating a large effect size.

**Conclusion:**

The mechanisms of the predictive effects of SCD-specific pain on generic HRQOL in pediatric patients with SCD are explained in part by the serial multiple mediator effects of sleep disturbance and general fatigue. Recognizing the multiple mediators of SCD-specific pain on generic HRQOL from the perspective of pediatric patients with SCD may aid future clinical research and practice to address impaired daily functioning through more comprehensive treatment approaches that include targeted symptom-specific interventions for pain, sleep disturbance, and fatigue.

## 1 Introduction

Pain has characteristically been considered the hallmark symptom of sickle cell disease (SCD), an inherited disease notably manifested by chronic hemolytic anemia and significantly impaired overall generic (non-disease-specific) health-related quality of life (HRQOL) (1–4). Although vaso-occlusive episodes are typically acknowledged as the source of severe acute pain in pediatric patients with SCD (5, 6), chronic and persistent pain has increasingly been identified as a substantial symptom burden for pediatric patients with SCD (7–11). In addition, fatigue and disturbed sleep patterns such as difficulty falling asleep at night or staying asleep throughout the night are also receiving heightened empirical attention in pediatric patients with SCD, given the potential negative impact of these persistent symptoms on HRQOL (12–14).

Patient-reported outcome (PRO) measurement of disease-specific symptoms and generic HRQOL are essential components for determining long-term patient outcomes in pediatric patients with SCD (2). PROs are reports derived directly from the patient perspective and include disease-specific and general symptoms that are known and felt only by patients themselves (15). Further, patient perspectives as measured by PROs are considered essential elements for shared clinical decision-making between pediatric patients with SCD and their healthcare providers (16).

While recent reviews have summarized the research on the purported mechanisms in the relationship between pain and sleep disturbances in general (17, 18), and the relationship between non-restorative sleep, fatigue, and HRQOL in young adults with autoimmune disease (19), unique to the current study, there has been no clinical research that has investigated pediatric patient self-reported sleep disturbance and general fatigue as serial (sequential) mediator variables that may explain in part the mechanisms of the predictive effects of SCD-specific pain on overall generic HRQOL of pediatric patients with SCD.

To address this important knowledge gap in the pediatric SCD empirical literature, perceived sleep disturbance and general fatigue are tested as serial mediator variables in a multiple mediator conceptual model that builds on our testing multiple mediators in pediatric patients with SCD pain (20), as well as our previous pain mediation conceptual models tested with other pediatric chronic health conditions (21–23). An understanding of the mechanisms by which SCD pain directly and indirectly predicts overall generic HRQOL may help identify particular clinical targets for patient healthcare as well as inform future clinical research for pediatric patients with chronic and recurrent SCD pain.

Specifically, the following serial multiple mediator model was investigated: pain → sleep disturbance → general fatigue → overall generic HRQOL. In this conceptual model, perceived sleep disturbance and general fatigue are hypothesized as sequential intervening variables in the relationship between SCD pain and overall generic HRQOL. In other words, SCD pain is hypothesized to result in more perceived sleep disturbance, which, in turn, is hypothesized to result in more general fatigue, all of which result in more impaired overall generic HRQOL in pediatric patients with SCD. Testing this conceptual model utilizes the existing database from the PedsQL Sickle Cell Disease Module field test study (24).

## 2 Methods

### 2.1 Participants and settings

Participants were pediatric patients who received a physician-confirmed diagnosis of SCD (any genotype) at five clinical centers across the United States as described in the PedsQL Sickle Cell Disease Module field test study (24). Parental informed consent and patient assent/consent were obtained. The human subjects’ institutional review boards at each center approved the study (24).

The participants for the present investigation include 227 pediatric patients aged 5–18 years from the field test study who completed the measures utilized in the current multivariate analyses. The average age of 104 boys (45.8%) and 123 girls (54.2%) out of the 227 was 11.53 years (SD = 3.88). With respect to race/ethnicity, the sample contained 221 (98.2%) self-reported Black non-Hispanics, 1 (0.4%) white non-Hispanic, 2 (0.9%) Hispanics, and 1 (0.4%) Other (missing = 2). The genotype included 161 (71.2%) HbSS, 45 (19.9%) HbSC, 18 (8.0%) HbS/beta-thalassemia, and 2 (0.8%) other rare genotypes (missing = 1). Additional demographic information is provided in the PedsQL Sickle Cell Disease Module field test study (24).

### 2.2 Measures

#### 2.2.1 PedsQL sickle cell disease module

The Pain and Hurt Scale (nine items, e.g., “I hurt all over my body”) from the PedsQL Sickle Cell Disease Module measures the SCD-specific pain construct developed through cognitive interviews with pediatric patients with SCD (25). The format, instructions, Likert response scale, and scoring method for the PedsQL Sickle Cell Disease Module scales are identical to those of the PedsQL 4.0 Generic Core Scales (26), with higher scores indicating better HRQOL and hence lower symptoms and problems. The instructions ask how much of a problem each item has been during the past one month, using the PedsQL 5-point Likert-type response scale (0 = never a problem; 1 = almost never a problem; 2 = sometimes a problem; 3 = often a problem; 4 = almost always a problem). To further increase the ease of use for the young child self-report (ages 5–7), the response scale is reworded and simplified to a 3-point scale (0 = not at all a problem; 2 = sometimes a problem; 4 = a lot of a problem). This simplification to a 3-point scale for the young child self-report is consistent with the PedsQL 4.0 Generic Core Scales as well as with all of the PedsQL disease-specific modules (27). Items are reverse-scored and linearly transformed to a 0–100 scale (0 = 100, 1 = 75, 2 = 50, 3 = 25, 4 = 0) so that lower scores demonstrate more SCD symptoms and problems and hence lower SCD-specific HRQOL.

#### 2.2.2 PedsQL multidimensional fatigue scale

The General Fatigue Scale (six items, e.g., “I feel too tired to do things that I like to do”) is contained in the PedsQL Multidimensional Fatigue Scale (12, 28, 29). The Sleep Disturbance Item (“It is hard for me to sleep through the night”) is derived from the Sleep/Rest Fatigue Scale contained in the PedsQL Multidimensional Fatigue Scale (12, 28, 29). This single item is chosen since it reflects most closely the concept of disturbed sleep in the Sleep/Rest Fatigue Scale. The PedsQL Multidimensional Fatigue Scale General Fatigue Scale has previously been shown to demonstrate acceptable internal consistency reliability and known-groups construct validity in pediatric patients with SCD (12). The single item “It is hard for me to sleep through the night” used in the present study also demonstrates known-groups construct validity using the legacy healthy controls sample from the previous study (12). Specifically, pediatric patients with SCD demonstrate a more than 20-point difference (61.89 vs. 83.37) in greater sleep disturbance as measured by this item in comparison with the legacy healthy controls [*t*(444) = 7.20, *P* < 0.001]. The format, instructions, Likert scale, and scoring method are identical to those of the PedsQL 4.0 Generic Core Scales so that lower scores demonstrate more symptoms.

#### 2.2.3 PedsQL 4.0 generic core scales

The 23-item PedsQL 4.0 Generic Core Scales encompass the following: (1) Physical Functioning (eight items), (2) Emotional Functioning (five items), (3) Social Functioning (five items), and (4) School Functioning (five items) (26). The Total Scale Score is comprised of the composite of the scores of the Physical, Emotional, Social, and School Functioning Scales. The Generic Core Scales Total Scale Score measures overall generic HRQOL (26). Higher scores indicate better HRQOL.

### 2.3 Statistical analysis

Pearson product-moment correlation analyses tested the bivariate associations between the perceived pain, sleep disturbance, and general fatigue variables with the Generic Core Scales Total Scale Score. Bivariate correlation effect sizes are designated as small (0.10), medium (0.30), and large (0.50) in magnitude (30). Hierarchical multiple regression analyses were conducted to statistically predict overall generic HRQOL by the perceived pain, sleep disturbance, and general fatigue variables after controlling for the factors of age and sex (31). Age and sex, but not race/ethnicity, were significantly associated in univariate analyses with at least one of the variables in the conceptual model for this database and were entered as demographic covariates in the multivariate analyses. Hierarchical multiple regression analyses tested the change in the variance accounted for by SCD pain in Step 2 and sleep disturbance and general fatigue in Step 3 (*R*^2^ changes) after controlling for age and sex (coded male = 1, female = 2) in Step 1. *R*^2^ values were reported for each step and the full model. Total *R*^2^ is the percentage of variability in the dependent variable (generic HRQOL) explained by the full model (demographic covariates, predictor, and mediators). *R*^2^ effect sizes are designated as small (0.02), medium (0.13), and large (0.26) in magnitude (30). These statistical analyses were conducted using IBM SPSS Statistics 29 (Armonk, New York).

Mediators are intervening variables hypothesized to account in part for the relationship between a predictor variable and an outcome variable (32, 33). The predictor variable is hypothesized to have a direct effect on the outcome variable, as well as an indirect effect through mediator variables. A serial multiple mediator model (34) was tested with perceived sleep disturbance and general fatigue as the hypothesized sequential mediators. It was hypothesized that greater SCD pain would result in greater sleep disturbance, which would result in greater general fatigue and together would result in more impaired overall generic HRQOL. The following serial multiple mediator model was tested: pain → sleep disturbance → general fatigue → overall generic HRQOL. Indirect (mediator) effects were tested utilizing 10,000 bias-corrected bootstrapped resamples with replacement yielding 95% confidence intervals. Significant indirect effects are demonstrated when the 95% confidence intervals do not include zero (34). These analyses were conducted using the PROCESS macro for IBM SPSS Statistics (processmacro.org) as described in Hayes (35).

## 3 Results

### 3.1 Pain predictor, mediators, and generic HRQOL means, standard deviations, and bivariate intercorrelations

Table 1 contains the pain predictor, mediators (sleep disturbance and general fatigue) with generic HRQOL (Generic Core Scales Total Scale Score) means, standard deviations, and bivariate intercorrelations. The SCD pain predictor and the sleep disturbance and general fatigue mediator variables were all significantly correlated with the Generic Core Scales Total Scale Score (all *P*s < 0.001), demonstrating large effect sizes.

**Table 1:** PedsQL Sickle Cell Disease Module Pain Scale, Multidimensional Fatigue Scale Sleep Disturbance Item and General Fatigue Scale, and Generic Core Scales Total Scale Score means and bivariate intercorrelations.

Item and scales	Items	*α*	Mean	SD	*r* * ^,a^	*r* * ^,b^	*r* * ^,c^
Pain	9	0.86	66.63	21.01	0.25*	0.50*	0.47*
Sleep Disturbance	1	__	61.89	34.91	__	0.54*	0.54*
General Fatigue	6	0.84	65.43	22.87	__	__	0.75*
Generic Core Total Scale Score	23	0.93	66.48	19.84	__	__	__

SD, standard deviation; *α*, Cronbach's alpha internal consistency reliability; *r*, Pearson product-moment correlation coefficient; Dashed line, not applicable.

Effect sizes for Pearson *r* designated as small (0.10), medium (0.30), and large (0.50) in magnitude.

Lower scores demonstrate worse symptoms and lower health-related quality of life.

a Bivariate correlations with Sleep Disturbance.

b Bivariate correlations with General Fatigue.

c Bivariate correlations with the Generic Core Scales Total Scale Score.

* All *P*s < 0.001.

### 3.2 Hierarchical multiple regression analysis predicting generic HRQOL

A hierarchical multiple regression analysis was conducted to analyze the percentage of the variance accounted for in the Generic Core Scales Total Scale Score by the SCD-specific pain predictor variable and the sleep disturbance and general fatigue mediator variables after controlling for age and sex. As demonstrated in Table 2, SCD-specific pain accounted for 23% of the variability in pediatric patient–reported generic HRQOL in Step 2, after accounting for age and sex in Step 1. The sleep disturbance and general fatigue mediator variables as a group accounted for an additional 39% of the variability in pediatric patient–reported generic HRQOL in Step 3, after accounting for the demographic covariates and SCD-specific pain predictor variable in Steps 1 and 2, respectively (*R*^2^ change, Table 2). *R*^2^ change for the sleep disturbance and general fatigue mediator variables as a group was statistically significant (*P* < 0.001), reflecting a large effect size.

**Table 2:** Hierarchical multiple regression analysis predicting PedsQL Generic Core Scales Total Scale Score by the pain predictor variable and sleep and general fatigue mediator variables controlling for age and sex.

Predictor and mediator variables	Regression values
Step 1. Demographic covariates	
Age (β )	0.02
Sex (β)	−0.01
*R*^2^	0.00
Step 2. Predictor variable	
Pain (β)	0.49*
*R*^2^ change	0.23*
Step 3. Mediator variables	
Sleep disturbance (β)	0.19*
General fatigue (β)	0.59*
*R*^2^ change	0.39*
*R*^2^ full model	0.63*

β, standardized regression coefficients (beta weights).

Sex coded: 1, male; 2, female.

*R*^2^, percentage of variability in the dependent variable (HRQOL) explained by the step.

*R*^2^ effect sizes designated as small (0.02), medium (0.13), and large (0.26).

* All *P*s < 0.001.

### 3.3 Serial multiple mediator model predicting generic HRQOL

**Figure 1:**
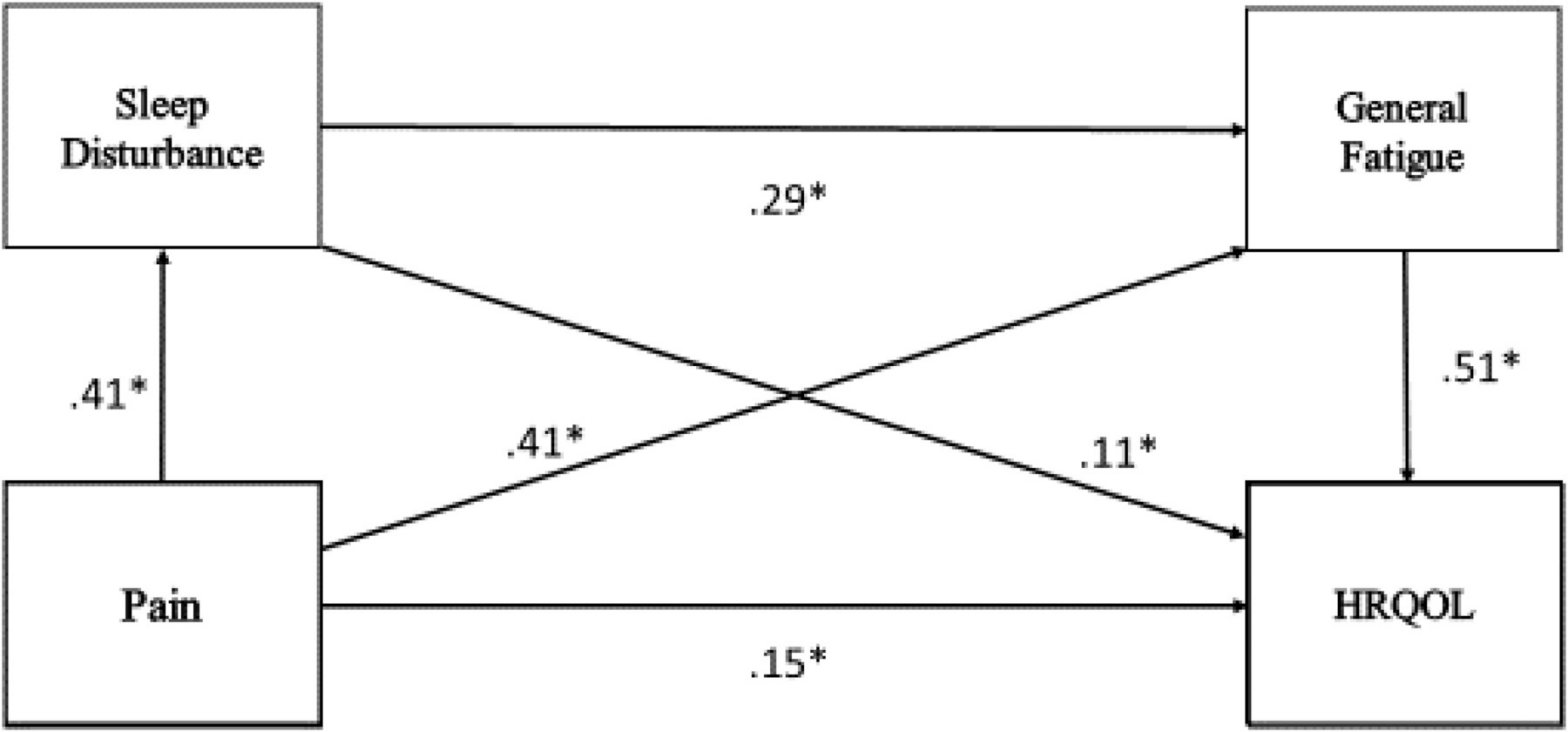
Hypothesized direct and indirect (mediator variables) effects of pain, sleep disturbance, and general fatigue on overall generic health-related quality of life (HRQOL). *All path coefficients are significant at *P* < 0.002.

After controlling for the age and sex covariates, the serial multiple mediator model demonstrated that the total indirect effect of the SCD-specific pain predictor variable on overall generic HRQOL as estimated by the sum of the indirect effects for sleep disturbance and general fatigue was 0.3179 and different from zero as determined by the bias-corrected bootstrap 95% confidence intervals that were above zero (0.2244, 0.4221). Within the multiple mediator model, the serial indirect effects for pain → sleep disturbance → general fatigue → generic HRQOL were 0.0614, and the bias-corrected bootstrap 95% confidence intervals did not contain zero (0.0280, 0.1075). The full serial multiple mediator model accounted for 63% of the variance in overall generic HRQOL (*P* < 0.001), demonstrating a large effect size (see Table 2). Figure 1 contains the specific path coefficients utilizing the Hayes Model 6 statistical diagram configuration for two mediators (p. 145) (35). All path coefficients were significant at *P* < 0.002. Figure 1 also demonstrates the significant direct effects of the pain predictor variable on the sleep disturbance and general fatigue mediator variables.

## 4 Discussion

The study results support the conceptual model that sleep disturbance and general fatigue serially mediate SCD-specific pain predictive effects on overall generic HRQOL in pediatric patients with SCD. In the multiple mediator model, sleep disturbance and general fatigue accounted for an additional 39% of the variance in overall generic HRQOL. The full serial multiple mediator conceptual model comprising age and sex demographic covariates, SCD-specific pain predictor variable, and the sleep disturbance and general fatigue mediator variables accounted for 63% of the variance in overall generic HRQOL in pediatric patients with SCD, reflecting a large effect size.

The serial multiple mediator findings further elucidate the multifaceted mechanisms in the connection between SCD-specific pain and overall generic HRQOL, suggesting symptom-specific intervention targets to reduce the symptom burden in pediatric patients with SCD and subsequently improve overall functioning and wellbeing. Thus, in addition to interventions targeting SCD-related pain (36, 37), sleep-promoting interventions focused on pediatric patient sleep disturbance and multicomponent general fatigue management strategies such as cognitive behavioral therapy and prescribed therapeutic exercise regimens may be adapted for pediatric patients with SCD (38, 39). Theoretically, such a multifaceted intervention approach may result in a greater enhancement of overall generic HRQOL for these young patients. Such a more comprehensive multifaceted treatment approach combining pain, sleep disturbance, and general fatigue interventions has not yet been empirically evaluated in pediatric patients with SCD with impaired overall generic HRQOL. As noted in the present study, the Sleep Disturbance Item score of 61.89 is more than 20 points lower (worse) than the item score for the legacy health controls (83.37) (12). In addition, the General Fatigue Scale score of 65.43 in the present study is 20 points lower (worse) than the previously published 86.1 score for the legacy healthy controls (12), while the Generic Core Scales Total Scale score of 66.48 is more than 17 points lower (worse) than the published PedsQL 4.0 Generic Core Scales score of 83.84 for healthy populations (40). Taken together, these findings clearly indicate the crucial need for more comprehensive treatment approaches for pediatric patients with SCD that incorporate pain, sleep disturbance, and fatigue symptom management interventions in order to more fully address the significantly impaired overall generic HRQOL of pediatric patients with SCD pain.

### 4.1 Strengths and limitations

The strengths of the study encompass testing a unique serial multiple mediator model specifically for pediatric patients with SCD, multisite recruitment, and large sample size. Limitations comprise absence of information on characteristics of individuals who declined participation and specific medical and treatment information on the existing field test database. Furthermore, cross-sectional analyses limit assumptions of directionality. Future studies with longitudinal analyses are needed to test the directionality of predictors and mediators (9, 41, 42). Future research would also benefit from parent proxy report for younger age participants who are not able to self-report. Finally, the Sleep Disturbance Item from the PedsQL Multidimensional Fatigue Scale is a pediatric patient self-report measure of one aspect of perceived sleep disturbance, which is, difficulty sleeping through the night. Previous studies have also used a single item to measure self-reported sleep in pediatric SCD (43, 44). Nevertheless, future conceptual models and clinical intervention research in pediatric patients with SCD pain will benefit from the inclusion of more comprehensive measures of disturbed sleep and sleep quality, as well as objective measures of disrupted sleep patterns (45, 46).

## 5 Conclusion

The mechanisms of the predictive effects of SCD-specific pain on overall generic HRQOL in pediatric patients with SCD are explained in part by the serial multiple mediator effects of sleep disturbance and general fatigue. Recognizing the multiple mediators of SCD-specific pain on overall generic HRQOL from the perspectives of pediatric patients with SCD may aid future clinical research and practice to address more fully their impaired daily functioning through more comprehensive treatment approaches that include targeted symptom-specific interventions for pain, sleep disturbance, and fatigue.

## Data Availability

The data are not publicly available due to privacy or ethical restrictions. Further queries can be directed to the corresponding author.

## References

[1] Kato GJ Piel FB Reid CD Gaston MH Ohene-Frempong K Krishnamurti L Sickle cell disease. Nat Rev Dis Primers. (2018) 4:18010. 10.1038/nrdp.2018.10 29542687

[2] Panepinto JA Bonner M. Health-related quality of life in sickle cell disease: past, present, and future. Pediatr Blood Cancer. (2012) 59:377–85. 10.1002/pbc.24176 22522407

[3] Dampier C Lieff S Lebeau P Rhee S McMurray M Rogers Z Health-related quality of life in children with sickle cell disease: a report from the comprehensive sickle cell centers clinical trial consortium. Pediatr Blood Cancer. (2010) 55:485–94. 10.1002/pbc.22497 20658620 PMC2911637

[4] Alberts NM Kang G Li C Richardson PA Hodges J Hankins JS Pain in youth with sickle cell disease: a report from the sickle cell clinical research and intervention program. Clin J Pain. (2021) 37:43–50. 10.1097/AJP.0000000000000889 33093339

[5] Brandow AM Brousseau DC Pajewski NM Panepinto JA. Vaso-occlusive painful events in sickle cell disease: impact on child well-being. Pediatr Blood Cancer. (2010) 54:92–7. 10.1002/pbc.22222 19653296 PMC3114448

[6] Rees CA Brousseau DC Ahmad FA Bennett J Bhatt S Bogie A Adherence to NHLBI guidelines for the emergent management of vaso-occlusive episodes in children with sickle cell disease: a multicenter perspective. Am J Hematol. (2022) 97:E412–5. 10.1002/ajh.26696 36054566 PMC9561082

[7] Sil S Cohen LL Dampier C. Psychosocial and functional outcomes in youth with chronic sickle cell pain. Clin J Pain. (2016) 32:527–33. 10.1097/AJP.0000000000000289 26379074

[8] Schlenz AM Schatz J McClellan CB Roberts CW. Responsiveness of the PedsQL to pain-related changes in health-related quality of life in pediatric sickle cell disease. J Pediatr Psychol. (2012) 37:798–807. 10.1093/jpepsy/jss051 22467881 PMC3404453

[9] Sil S Cohen LL Bakshi N Watt A Hathaway M Abudulai F Changes in pain and psychosocial functioning and transition to chronic pain in pediatric sickle cell disease: a cohort follow-up study. Clin J Pain. (2020) 36:463–71. 10.1097/AJP.0000000000000827 32287106 PMC7233325

[10] Sil S Manikowski A Schneider M Cohen LL Dampier C. Identifying chronic pain subgroups in pediatric sickle cell disease: a cluster-analytic approach. Clin J Pain. (2022) 38:601–11. 10.1097/AJP.0000000000001065 35997659 PMC9481686

[11] Smith WR McClish DK Valrie C Sisler I. What interval of daily pain assessment is required to reliably diagnose chronic pain in SCD? The pain in sickle cell epidemiology study. J Sick Cell Dis. (2024) 1:yoae011. 10.1093/jscdis/yoae011 40304011 PMC12039818

[12] Panepinto JA Torres S Bendo CB McCavit TL Dinu B Sherman-Bien S PedsQL™ multidimensional fatigue scale in sickle cell disease: feasibility, reliability and validity. Pediatr Blood Cancer. (2014) 61:171–7. 10.1002/pbc.24776 24038960 PMC3848797

[13] Anderson LM Allen TM Thornburg CD Bonner MJ. Fatigue in children with sickle cell disease: association with neurocognitive and social-emotional functioning and quality of life. J Pediatr Hematol Oncol. (2015) 37:584–9. 10.1097/MPH.0000000000000431 26479993

[14] Kölbel M Kirkham FJ Dimitriou D. Developmental profile of sleep and its potential impact on daytime functioning from childhood to adulthood in sickle cell anaemia. Brain Sci. (2020) 10(12):981. 10.3390/brainsci10120981 33327459 PMC7764980

[15] FDA. Guidance for Industry: Patient-Reported Outcome Measures: Use in Medical Product Development to Support Labeling Claims. Rockville, MD: Food and Drug Administration, US Department of Health and Human Services (2009).

[16] Beverung LM Varni JW Panepinto JA. Clinically meaningful interpretation of pediatric health-related quality of life in sickle cell disease. J Pediatr Hematol Oncol. (2015) 37:128–33. 10.1097/MPH.0000000000000177 24942019 PMC4269583

[17] Whibley D AlKandari N Kristensen K Barnish M Rzewuska M Druce KL Sleep and pain: a systematic review of studies of mediation. Clin J Pain. (2019) 35:544–58. 10.1097/AJP.0000000000000697 30829737 PMC6504189

[18] Babiloni H De Koninck A Beetz BP De Beaumont G Martel L & Lavigne MO Sleep and pain: recent insights, mechanisms, and future directions in the investigation of this relationship. J Neural Transm. (2020) 127:647–60. 10.1007/s00702-019-02067-z 31452048

[19] Fletcher-Sandersjöö S Westeinde AVT Spelman T Hirvikosi T Lajic S Bensing S. The relationship between sleep, fatigue and quality of life in young adults with autoimmune Addison’s disease. *Clin Endocrinol (Oxf)*. (in press). 10.1111/cen.70028 PMC12583308 40891960

[20] Varni JW Panepinto JA. Cognitive functioning, patient health communication, and worry mediate pain predictive effects on health-related quality of life in youth with sickle cell disease. Pediatr Blood Cancer. (2020) 67:e28680. 10.1002/pbc.28680 32860648

[21] Varni JW Nutakki K Swigonski NL. Cognitive functioning and pain interference mediate pain predictive effects on health-related quality of life in pediatric patients with Neurofibromatosis Type 1. Eur J Paediatr Neurol. (2020) 28:64–9. 10.1016/j.ejpn.2020.07.014 32847704

[22] Varni JW Uzark K. Pain and health-related quality of life in Duchenne muscular dystrophy: a multiple mediator analysis. Eur J Paediatr Neurol. (2023) 46:61–6. 10.1016/j.ejpn.2023.07.003 37463545

[23] Varni JW Zebracki K Hwang M Mulcahey MJ Vogel LC. Pain, pain interference, social and school/work functioning in youth with spinal cord injury: a mediation analysis. J Spinal Cord Med. (2024) 47:504–10. 10.1080/10790268.2022.2120232 36149340 PMC11218589

[24] Panepinto JA Torres S Bendo CB McCavit TL Dinu B Sherman-Bien S PedsQL™ sickle cell disease module: feasibility, reliability and validity. Pediatr Blood Cancer. (2013) 60:1338–44. 10.1002/pbc.24491 23441057 PMC4412167

[25] Panepinto JA Torres S Varni JW. Development of the PedsQL™ sickle cell disease module items: qualitative methods. Qual Life Res. (2012) 21:341–57. 10.1007/s11136-011-9941-4 21638090 PMC3277645

[26] Varni JW Seid M Kurtin PS. PedsQL™ 4.0: reliability and validity of the pediatric quality of life inventory™ version 4.0 generic core scales in healthy and patient populations. Med Care. (2001) 39:800–12. 10.1097/00005650-200108000-00006 11468499

[27] Varni JW Limbers CA. The pediatric quality of life inventory: measuring pediatric health-related quality of life from the perspective of children and their parents. Pediatr Clin N Am. (2009) 56:843–63. 10.1016/j.pcl.2009.05.016 19660631

[28] Varni JW Burwinkle TM Katz ER Meeske K Dickinson P. The PedsQL™ in pediatric cancer: reliability and validity of the pediatric quality of life inventory™ generic core scales, multidimensional fatigue scale, and cancer module. Cancer. (2002) 94:2090–106. 10.1002/cncr.10428 11932914

[29] Varni JW Beaujean A Limbers CA. Factorial invariance of pediatric patient self-reported fatigue across age and gender: a multigroup confirmatory factor analysis approach utilizing the PedsQL™ multidimensional fatigue scale. Qual Life Res. (2013) 22:2581–94. 10.1007/s11136-013-0370-4 23423759

[30] Cohen J. Statistical Power Analysis for the Behavioral Sciences, 2nd ed. Hillsdale, NJ: Erlbaum (1988).

[31] Cohen J Cohen P West SG Aiken LS. Applied Multiple Regression/Correlation Analysis for the Behavioral Sciences, 3rd ed. Mahwah, NJ: Erlbaum (2003).

[32] Baron RM Kenny DA. The moderator-mediator variable distinction in social psychological research: conceptual, strategic, and statistical considerations. J Pers Soc Psychol. (1986) 51:1173–82. 10.1037/0022-3514.51.6.1173 3806354

[33] Preacher KJ Hayes AF. Asymptotic and resampling strategies for assessing and comparing indirect effects in multiple mediator models. Behav Res Methods. (2008) 40:879–91. 10.3758/BRM.40.3.879 18697684

[34] Hayes AF Rockwood NJ. Regression-based statistical mediation and moderation analysis in clinical research: observations, recommendations, and implementation. Behav Res Ther. (2017) 98:39–57. 10.1016/j.brat.2016.11.001 27865431

[35] Hayes AF. Introduction to Mediation, Moderation, and Conditional Process Analysis: A Regression-Based Approach. New York, NY: Guilford (2013).

[36] Sil S Lai K Lee JL Gilleland Marchak J Thompson B Cohen L Preliminary evaluation of the clinical implementation of cognitive-behavioral therapy for chronic pain management in pediatric sickle cell disease. Complement Ther Med. (2020) 49:102348. 10.1016/j.ctim.2020.102348 32147059 PMC7092728

[37] Palermo TM Lalloo C Zhou C Dampier C Zempsky W Badawy SM A cognitive-behavioral digital health intervention for sickle cell disease pain in adolescents: a randomized, controlled, multicenter trial. Pain. (2024) 165:164–76. 10.1097/j.pain.0000000000003009 37733479 PMC10723646

[38] Griggs S Conley S Batten J Grey M. A systematic review and meta-analysis of behavioral sleep interventions for adolescents and emerging adults. Sleep Med Rev. (2020) 54:101356. 10.1016/j.smrv.2020.101356 32731152 PMC7669566

[39] Moss-Morris R Harrison AM Safari R Norton S van der Linden ML Picariello F Which behavioural and exercise interventions targeting fatigue show the most promise in multiple sclerosis? A systematic review with narrative synthesis and meta-analysis. Behav Res Ther. (2021) 137:103464. 10.1016/j.brat.2019.103464 31780252

[40] Varni JW Limbers CA Burwinkle TM. Impaired health-related quality of life in children and adolescents with chronic conditions: a comparative analysis of 10 disease clusters and 33 disease categories/severities utilizing the PedsQL™ 4.0 generic core scales. Health Qual Life Outcomes. (2007) 5(43):1–15. 10.1186/1477-7525-5-1 17634123 PMC1964786

[41] Panepinto JA Scott JP Badaki-Makun O Darbari DS Chumpitazi CE Airewele GE Determining the longitudinal validity and meaningful differences in HRQL of the PedsQL™ sickle cell disease module. Health Qual Life Outcomes. (2017) 15(1):124. 10.1186/s12955-017-0700-2 28606098 PMC5468970

[42] Karlson CW Barajas KG Seals SR Britt AB Schlenz AM Jackson EA Longitudinal predictors of pain in pediatric sickle cell disease. J Pediatr Psychol. (2023) 48:553–61. 10.1093/jpepsy/jsad017 37043758

[43] Valrie CR Gil KM Redding-Lallinger R Daeschner C. Daily mood as a mediator or moderator of the pain-sleep relationship in children with sickle cell disease. J Pediatr Psychol. (2008) 33:317–22. 10.1093/jpepsy/jsm058 17690117

[44] Johnston JD Reinman LC Bills SE Schatz JC. Sleep and fatigue among youth with sickle cell disease: a daily diary study. J Behav Med. (2023) 46:440–50. 10.1007/s10865-022-00368-5 36334167 PMC9638215

[45] Fisher K Laikin AM Sharp KMH Criddle CA Palermo TM Karlson CW. Temporal relationship between daily pain and actigraphy sleep patterns in pediatric sickle cell disease. J Behav Med. (2018) 41:416–22. 10.1007/s10865-018-9918-7 29532199

[46] Giordano NA Getz T Gottschalk M Miller AH Dupree Jones K Park J Sleep hygiene linked to patient-reported outcomes & objective sleep measures prior to upper extremity orthopaedic surgery. Front Pain Res. (2025) 6:1589748. 10.3389/fpain.2025.1589748 PMC12187727 40568187

